# Stressful Events and Continued Smoking and Continued Alcohol Consumption during Mid-Pregnancy

**DOI:** 10.1371/journal.pone.0086359

**Published:** 2014-01-20

**Authors:** Chantal Beijers, Johan Ormel, Judith L. Meijer, Tjitte Verbeek, Claudi L. H. Bockting, Huibert Burger

**Affiliations:** 1 University of Groningen, University Medical Center Groningen, Interdisciplinary Center of Psychopathology and Emotion regulation, Groningen, The Netherlands; 2 University of Groningen, University Medical Center Groningen, Department of Epidemiology, Groningen, The Netherlands; 3 University of Groningen, Department of Clinical Psychology, Groningen, The Netherlands; 4 University of Groningen, University Medical Center Groningen, Department of General Practice, Groningen, The Netherlands; Erasmus University Rotterdam, Netherlands

## Abstract

**Aim:**

to examine whether the severity of different categories of stressful events is associated with continued smoking and alcohol consumption during mid-pregnancy. Also, we explored the explanation of these associations by anxiety and depressive symptoms during pregnancy. Finally, we studied whether the severity of stressful events was associated with the amount of cigarettes and alcohol used by continued users.

**Method:**

we conducted a cross-sectional analysis using data from a population-based prospective cohort study. Pregnant women were recruited via midwifery practices throughout The Netherlands. We analyzed women who continued smoking (*n = 113*) or quit (*n = 290*), and women who continued alcohol consumption (*n = 124)* or quit (*n = 1403)* during pregnancy. Smoking, alcohol consumption, and perceived severity of stressful events were measured at 19 weeks of gestation. The State Trait Anxiety Inventory and the Edinburgh Postnatal Depression Scale were filled out at 14 weeks of gestation. Odds ratios were calculated as association measures and indicated the relative increase for the odds of continuation of smoking and alcohol consumption for the maximum severity score compared to the minimum score.

**Findings:**

severity of the following stressful event categories was associated with continued alcohol consumption: ‘conflict with loved ones’ (OR = 10.4, p<0.01), ‘crime related’ (OR = 35.7, p<0.05), ‘pregnancy-specific’ (OR = 13.4, p<0.05), and the total including all events (OR = 17.2, p<0.05). Adjustment for potential confounders (age, parity and educational level) did not notably change the estimates. There was no association of anxiety and depressive symptoms with continued smoking or alcohol consumption. No associations emerged for continued smoking and severity of stressful events. The amount of cigarettes and alcohol consumption among continued users was not associated with severity of stressful events.

**Conclusions:**

Our findings may be relevant for health care providers, in particular midwives and general practitioners. The impact of stressful events may be considered when advising pregnant women on smoking and alcohol consumption.

## Introduction

The hazardous effects of smoking and alcohol consumption during pregnancy are well-acknowledged. For smoking these include preterm birth, low birth weight, lower Apgar scores [1], sudden infant death syndrome [2], [3], changes in brain development [4], [5], increased risk for obesity in adolescence [6] and even behavioral problems in the long term [7]. Alcohol consumption during pregnancy has been associated with preterm delivery [8], spontaneous abortion [9], reduced birth weight [10], the fetal alcohol syndrome [11] and the child’s IQ score at age 8 [12]. Nevertheless, smoking prevalence rates during pregnancy range from 5% to 21% [13], [14] and alcohol consumption prevalence rates vary between 6% and 50% in western countries [15]–[20].

Among the suggested risk factors for continuation of smoking and alcohol consumption during pregnancy are a low educational level, being single, being multiparae, and high perceived psychological stress in early pregnancy [21]–[24]. As certain events in life can generate psychological stress [25], they may be associated with continued smoking and alcohol consumption during pregnancy. Surprisingly, not much is known about continued versus quit smoking and alcohol consumption associated with stressful events during pregnancy. Only a single previous study has examined stressful events in pregnancy and continued versus quit smoking. No association with the number of stressful events was found [21]. Yet, perceived severity of events was not taken into account. Also, no distinction was made between different categories of stressful events. In particular, pregnancy-specific stressful events such as finding out about congenital anomalies or experiencing alarming obstetric symptoms (e.g. vaginal bleeding) may be especially relevant during pregnancy. As for continued versus quit alcohol consumption, and the association with stressful events during pregnancy, no study has been undertaken, to the best of our knowledge. Some insight into the explanation of the association between stressful events and continued smoking and alcohol consumption during pregnancy may be obtained by taking antenatal symptoms of anxiety and depression into account. Several studies showed that smoking and alcohol consumption during pregnancy are related to anxiety and depression symptoms [26]–[28], and stressful events predict these symptoms [29], [30]. Therefore, these symptoms may explain part of the association between stressful events and continued smoking and alcohol consumption during pregnancy. To our knowledge, this explanation has not been researched to date.

In the present study we examined the associations of perceived severity of different categories of stressful events, including pregnancy-specific events, with continued smoking and continued alcohol consumption during mid-pregnancy. We hypothesized that increased perceived severity of events would be associated with continued smoking and alcohol consumption. Perceived severity of events was assessed using a subjective and normative approach. We further explored whether some proportion of the associations between severity of stressful events and continued smoking and alcohol consumption would be explained by anxiety or depressive symptoms. In addition, we assumed that with increasing severity of events the amount of smoking and alcohol consumption among continued users would be higher.

## Methods

### Setting and Participants

The present cross-sectional analysis was performed within the Pregnancy, Anxiety and Depression (PAD) study [31]. This ongoing population-based prospective cohort study investigates psychological, medical and social factors during pregnancy and in the postnatal period. Participants are enrolled at primary midwifery practices (n = 102) and obstetric and gynecology departments of hospitals (n = 9) throughout The Netherlands. Women who provide written informed consent enter the study in the first trimester of pregnancy. After two baseline measurements at approximately 14 and 19 weeks estimated gestational age (EGA), follow-up assessments using online questionnaires take place up to 6 months after pregnancy. For the present analysis, data was used from all participants that completed baseline measurements between December 2011 and July 2013. Out of 3383 women that agreed on participating in that period, 2287 (68%) women completed the baseline assessments. The PAD study was approved by the medical ethical review board of the University Medical Center Groningen.

### Smoking Status

Current smoking status and smoking status before pregnancy were ascertained by self-report during mid-pregnancy, i.e. at the 19-week EGA baseline measurement, using the following two questions: (1) ‘*Did you smoke cigarettes before finding out about your current pregnancy*?’ (yes/no) and (2) ‘*Are you currently smoking cigarettes*?’ (yes/no). “Continued smoking” was defined as a positive response to both questions 1 and 2. “Quit smoking” was defined as a positive response to question 1 and a negative response to question 2. “Not smoking” was defined as a negative response to both questions. We asked continued smokers to categorize their typical average amount of cigarettes smoked per day using five classes: 1–5, 6–10, 11–15, 16–20 and 21 or more.

### Alcohol Consumption Status

Current alcohol consumption status and alcohol consumption status before pregnancy were ascertained by self-report during mid-pregnancy, i.e. at the 19-week EGA baseline measurement, using the following two questions: 1) *‘Did you drink alcohol before finding out about your current pregnancy?’* (yes/no) and 2) *‘How often do you drink during the week?*’. “Continued alcohol consumption” was defined as a positive response to question 1 and a response larger than zero to question 2. “Quit alcohol consumption” was defined as a positive response to question 1 and a zero-response to question 2. “Not drinking alcohol” was defined as a negative response to question 1 and a zero-response to question 2. Typical frequency of alcohol consumption among continued users was assessed in the following seven categories: less than once a month, once a month, 2 to 3 times a month, and 1, 2, 3, 4 or more day(s) per week. The typical amount of alcohol consumption was assessed using five categories: 1–2, 3–5, 6–10, 11–15, and over 15 glasses each time. Frequency of alcohol consumption was multiplied by the amount to provide a single estimate of the mean total amount of weekly alcohol consumption.

### Stressful Events

Stressful events during early and mid-pregnancy were assessed at the 19-week EGA baseline measurement using 47 translated events from the event questionnaire developed and utilized in the Avon Longitudinal Study of Parents And Children (ALSPAC study) [32]. Event items consist of different categories of events (e.g. events related to work, financial issues, family, crime), including pregnancy-specific events. Participants were asked to confirm whether these events had occurred during pregnancy and were asked about their perceived severity. Severity was rated as scores: “affected me a lot (4), affected me moderately (3), affected me mildly (2) and did not affect me at all (1)”. All stressful events were divided into nine core event categories: work or study related events of self or partner, financial related events, events related to conflict with loved ones, events related to housing, events related to death of loved ones, events related to illness of self or loved ones, events related to domestic violence or abuse, crime related events, and pregnancy-specific events (table S1). To account for the severity of events, a subjective and a normative approach was followed. We followed both approaches to account for response-tendency due to large variations that may exist in the appraisal of event severity, making comparisons between participants difficult [33]. Correspondingly, two types of severity weights were calculated for each event. The individual severity weight of an event is the individual-specific rating and thus varies across participants. In contrast, and analogous to the approach followed by Holmes and Rahe in creating the social readjustment scale [34], the group severity weight of a certain event was defined as the severity experienced on the group level. This weight was calculated as the mean of all individual severity scores given for that event in our study population. This severity weight is therefore constant across participants (table S1). For each participant we calculated, for each event category separately, an individual severity sum (ISS) score by adding up all individual severity scores of events experienced. Likewise, a group severity sum score (GSS) was calculated. Finally, ISS and GSS scores were added up over the nine general categories to arrive at a total severity score. Some stressful events were considered ambiguous with regard to their unpleasantness, e.g. moving house. To distinct unpleasant events from ambiguous events, two raters (JM and TV) independently judged whether events were either predominantly unpleasant, or predominantly pleasant (yes/no).

### Anxiety and Depression

At the 14-week EGA baseline measurement, anxiety symptoms were measured using the Dutch version of the validated 6-item State Trait Anxiety Inventory [35], and depressive symptoms were measured using the Dutch version of the validated 10-item Edinburgh Postnatal Depression Scale [36].

### Other Variables

Age, educational level, and parity were considered potential confounders based on their association with continued smoking and alcohol consumption, and with stressful events [23], [37]. Educational level was recorded as elementary education, lower tracts of secondary education, higher tracts of secondary education, higher vocational education, or university education. Parity was assessed as primiparae or multiparae. Furthermore, we registered country of birth for descriptive purposes.

### Multiple Imputation of Missing Data

To avoid the risk of bias and loss of statistical power in complete-case analysis, missing data were imputed using multiple imputation by chained equations under the assumption that the missing data mechanism was missing at random (MAR) or missing completely at random. Multiple imputation is considered an appropriate method for dealing with missing data [38]. Following recommendations by Graham [39], 20 datasets were imputed and combined according to Rubin’s rules [40]. The percentage of missing data was approximately 25% for the variables of interest. The imputation model included all variables of interest including the outcome variables. For each of the variables we studied the missing data mechanism. This was done by predicting missingness (yes/no) of each of these variables from the other variables in the imputation model using a multivariable logistic regression analysis. These analyses showed explained variances ranging from 2.6% to 46.6% (Nagelkerke’s R^2^). This means that data were MAR at least to some extent, and consequently multiple imputation may have minimized bias. However, data being missing not at random can never be excluded. As a sensitivity analysis, we performed complete-case analyses (CCA).

### Data Analysis

We calculated descriptive statistics for the total study population according to smoking and alcohol consumption status. Subsequent analyses included continued users and quitters only. To that end, two groups were identified: those who either continued or quit smoking and those who either continued or quit alcohol consumption. The number of participants who continued both smoking and alcohol consumption was too small to study (n = 4). Groups were compared using t-tests, Mann-Whitney tests, Pearson Chi-Square tests where appropriate. Logistic regression analysis was used to investigate the associations of continued smoking (dependent variable) with severity of stressful events (independent variables). Likewise, continued alcohol consumption was included as dependent variable. Odds ratios (OR) from these analyses were calculated as quantitative association measures and were accompanied by 95% confidence intervals.

All ISS and GSS scores were entered as continuous variables and, to ensure comparability, were first standardized to a value ranging from 0 to 1 by division through their maximum value. Therefore, OR indicated the relative increase of the odds of continuation of smoking and alcohol consumption for the maximum compared to the minimum score. In separate analyses, ambiguous or predominantly pleasant events were excluded to specifically assess the outcome of unpleasant stressful events. In additional regression analyses, we adjusted for the potential confounders. Stability of the regression models that showed statistically significant results was checked by creating three severity score categories, based on the distribution of the ISS and GSS scores, and investigating them as an independent variable. As a supplementary, exploratory analysis, the associations between the occurrence of individual stressful events listed in table S1 and continued smoking and alcohol consumption was investigated, irrespective of their severity. Before exploring the explanation of the associations by anxiety or depressive symptoms, we first investigated whether these symptoms were associated with the dependent and independent variables. If so, these symptoms were added separately as independent continuous variables to the adjusted regression models. Resulting changes in beta-coefficients for event categories were considered measures of explanation. We beforehand decided that changes more than 10% indicated the presence of explanation [41]. We limited these analyses to those event categories that showed to be statistically significantly associated with continued smoking or alcohol consumption. Spearman’s rank correlation was used as a measure for the relationships between severity of stressful events, and the amount of cigarettes and alcohol. The level of statistical significance was set at 0.05, two-sided. Multiple imputation and all analyses were performed using IBM SPSS Statistics version 20.0.

## Results

### Descriptives

Table 1 presents the characteristics of the total study population after imputation. One hundred and thirteen (28.0%) participants continued smoking and 124 (8.1%) participants continued alcohol consumption. Continued smokers had a lower educational level (p = 0.02) and experienced one or more stressful event(s) during pregnancy more often compared to quitters, although this latter difference was not statistically significant (p = 0.08). Continued alcohol consumers were older compared to quitters (p = 0.03). Regarding the average amount of cigarettes smoked per day, 59 (52.2%) participants smoked 1–5, 35 (31.0%) smoked 6–10 and 19 (16.8%) smoked more than 10. Continued alcohol consumers drank on average one glass of alcohol per week. Table S1 shows for each stressful event its prevalence by smoking and alcohol consumption status. Prevalence rates of events related to domestic violence or abuse were low, therefore no associations were calculated for this category.

**Table 1:** Descriptive data (pooled) of study participants according to smoking and alcohol consumption status (n = 2287).

Characteristic	Continued smoking(n = 113)	Quit smoking(n = 290)	P-value	Not smoking(n = 1883)	Continued alcoholcons. (n = 124)	Quit alcohol cons.(n = 1403)	P-value	Not drinking alcohol (n = 760)
**Age (mean, SD)**	30.5 (5.6)	30.6 (4.9)	0.87	31.8 (4.4)	32.8 (4.4)	31.7 (4.5)	**0.03**	31.2 (4.7)
**Education (n, %)**			**0.02**				0.21	
Elementary education	3 (2.7)	4 (1.4)		4 (0.2)	0	5 (0.4)		5 (0.7)
Lower tracts of secondary education	23 (20.4)	51 (17.6)		126 (6.7)	9 (7.3)	115 (8.2)		78 (10.3)
Higher tracts of secondary education	61 (54.0)	108 (37.2)		462 (24.5)	21 (16.9)	341 (24.3)		269 (35.4)
Higher vocational education	22 (19.5)	93 (32.1)		778 (41.3)	50 (40.3)	557 (39.7)		286 (37.6)
University education	5 (4.4)	34 (11.7)		512 (27.2)	44 (35.5)	385 (27.4)		122 (16.1)
**Multiparous** **(n, %)**	63 (55.8)	154 (53.1)	0.64	1105 (58.7)	75 (60.5)	756 (53.9)	0.21	492 (64.7)
**≥1 event** **(n, %)**	104 (92.0)	246 (84.8)	0.08	1527 (81.1)	109 (87.9)	1170 (83.4)	0.23	598 (78.7)
**Anxiety score (median, IQR)**	36.5 (16.7)	34.1 (11.1)	0.08	33.3 (10.0)	33.3 (11.2)	33.3 (10.0)	0.15	33.3 (11.5)
**Depression score (median, IQR)**	5.4 (6.4)	5.0 (5.5)	0.18	4.0 (4.6)	4.5 (5.7)	4.0 (5.0)	0.20	4.0 (5.0)
**Country of birth**			0.35				0.17	
Netherlands	111 (97.3)	271 (93.4)		1787 (94.9)	114 (91.9)	1333 (95.0)		722 (95.0)
Maroc/Turkey/Suriname/Aruba	1 (0.9)	4 (1.4)		10 (0.5)	1 (0.8)	7 (0.5)		6 (0.8)
Other	1 (0.9)	15 (5.2)		87 (4.6)	8 (6.5)	63 (4.5)		32 (4.2)

P-values are given for continued versus quit use.

Note: for some variables numbers do not add to the total due to rounding of imputed values.

### Stressful Events and Continued Smoking

ISS and GSS scores of event categories showed a correlation >0.9, therefore ISS scores are reported only. ISS scores of event categories showed no statistical significant association with continued smoking (table 2). Adjustment for potential confounders only slightly weakened the associations. ‘Starting a new job’, ‘moving house’, and ‘being told to have twins’ were rated as not predominantly unpleasant by both raters. Exclusion of these events from the analyses did not markedly change the estimates. Individual stressful events were not associated with continued smoking (table S1). Results from the CCA were not notably different from the imputed results.

**Table 2:** Mean individual severity sum and total scores, and unadjusted odds ratios (OR) with 95% confidence intervals (95% CI) for the associations of individual severity sum scores and total scores with continuation of smoking and alcohol consumption.

	Continued smoking		Continued alcohol consumption	
Event category	Mean (min-max)	OR (95% CI)	Mean (min-max)	OR (95% CI)
Work or study related	1.9 (0–20)	1.5 (0.3;9.0)	1.5 (0–22)	3.5 (0.5;22.8)
Financially related	1.0 (0–8)	1.5 (0.6;4.0)	0.6 (0–8)	1.7 (0.6;4.9)
Conflict loved ones	1.3 (0–16)	1.8 (0.4;9.7)	0.7 (0–16)	10.4 (2.0;54.7)**
Housing related	0.3 (0–4)	1.9 (0.5;7.1)	0.2 (0–4)	1.5 (0.4;5.0)
Death of loved ones	0.7 (0–16)	3.9 (0.3;50.9)	0.5 (0–15)	2.0 (0.2;24.7)
Illness self or loved ones	1.3 (0–24)	2.3 (0.2;25.8)	1.1 (0–24)	0.6 (0.0;15.3)
Crime related	0.3 (0–17)	0.2 (0.0;16.0)	0.2 (0–17)	35.7 (1.8;711.0)*
Pregnancy-specific	2.1 (0–28)	1.2 (0.1;13.6)	1.9 (0–28)	13.4 (1.8;98.1)*
Total score	9.1 (0–104)	2.9 (0.2;40.3)	6.7 (0–104)	17.2 (1.3;235.6)*

Scores were standardized to a value ranging from 0 to 1 before entrance into logistic regression models as independent variables.

Estimates were not calculated for the category ‘domestic violence and abuse’ due to low prevalence rates.

p<0.05.

p<0.01.

### Stressful Events and Continued Alcohol Consumption

ISS and GSS scores of event categories showed a correlation >0.9, therefore ISS scores are reported only. ISS scores were statistically significantly associated with continued alcohol consumption in the following categories: ‘conflict with loved ones’, ‘crime related’, ‘pregnancy-specific’, and the total severity score (table 2). Adjustment did not notably affect the estimates. ISS scores of the category ‘pregnancy-specific’ and the total severity score showed an increase in OR with increasing category. Exclusion of events rated as not predominantly unpleasant did not noteworthy change the estimates. Individual stressful events that were associated with continued alcohol consumption consisted of: ‘arguing with partner’, ‘arguing with family’, ‘having an unwanted pregnancy’, and ‘taking a test to see if the baby might not be normal’ (p<0.05) (table S1). Results from the CCA were not notably different from the imputed results.

### Explanation by Anxiety and Depression Symptoms

Anxiety and depressive symptoms were not associated with continued smoking and alcohol consumption (table 1), therefore we refrained from the analyses that would investigate to what extent the associations could be explained by these symptoms.

### The Amount of Cigarettes and Alcohol Consumption during Pregnancy

Spearman’s correlation coefficient ranged between −0.05 and 0.27 for the associations of severity of stressful events with the amount of cigarettes and alcohol consumption. None of the correlations were statistically significant.

## Discussion

To our knowledge, this is the first study that investigated the associations between perceived severity of different categories of stressful events, including pregnancy-specific events, and continued versus quit smoking or alcohol consumption during mid-pregnancy. Our results showed that the total perceived severity of stressful events during early and mid-pregnancy was associated with continued alcohol consumption. Next to the total perceived severity, perceived severity of events in the following categories showed associations with continued alcohol consumption: ‘conflict with loved ones’, ‘crime related’, and ‘pregnancy-specific’. No associations emerged between severity of stressful events and continued smoking. Furthermore, the associations could not be explained by anxiety and depressive symptoms. The amount of cigarettes and alcohol consumption during pregnancy was not associated with severity of stressful events.

Our finding that the event category ‘conflict with loved ones’ was statistically significantly associated with continued alcohol consumption corroborates previous research. Support by and relationship with partner assists in dealing with stress, especially during pregnancy. Rosand and colleagues suggested that relationship satisfaction during pregnancy alone can act as a protective factor for handling certain stressors [42]. Social support is in general considered an important protective factor for coping with stressful events [43], [44], and especially the mother of pregnant women is an important source of social support during pregnancy [45]. In addition, our exploratory analyses of individual stressful events revealed that the occurrence of arguing with partner or family alone may increase the odds of continued alcohol consumption. The association we observed between perceived severity of pregnancy-specific events and continued alcohol consumption confirms that it may be relevant to distinguish this category of events in particular [46]–[48]. For example, Lobel and colleagues showed that pregnancy-specific stress was a stronger predictor of adverse birth outcomes than general stress [46].

Overall, our findings provide insights on the impact of different categories of stressful events, including pregnancy-specific events, and continued smoking and alcohol consumption during mid-pregnancy. Our results may be especially relevant for health care providers, such as midwifes or general practitioners, who advise women on smoking and alcohol consumption during pregnancy. The impact of stressful events should be discussed during pregnancy in the context of healthy lifestyle strategies. Pregnancy-specific events may even take place in the context of prenatal care (e.g. taking a test to see if your baby might not be normal), and their impact can therefore easily be addressed by health care professionals. The given that we were not able to demonstrate statistical significant findings on event categories and continued smoking suggests that the role of perceived severity of stressful events is limited compared to continued alcohol consumption. There may be other, more prominent factors that determine continued smoking during pregnancy. For example, a low socio-economic status, having a partner that smokes and great nicotine dependence have consistently been reported as barriers to smoking cessation [49]–[51].

We did not find evidence for our suggestion that the associations between perceived severity of events and continued alcohol consumption could be explained by anxiety and depressive symptoms. This may be because alcohol consumption is part of a coping mechanism involved in dealing with stressful events via a direct pathway or via other, unknown, factors. Arch found that pregnancy-specific anxiety was associated with alcohol consumption during pregnancy rather than general anxiety [52]. Therefore, our analyses could have yielded different results if we had measured pregnancy-specific anxiety.

The quitting rate found in our study was 72.0% for smoking and 91.9% for alcohol consumption, which are high rates when compared to previous studies [51], [53]–[55]. As earlier research shows that a low educational level is associated with continued smoking and alcohol consumption during pregnancy [23], [56], the relatively high quitting rates may be explained by the composition of our study population that included relatively few lower educated women.

Despite the large total sample, the relatively small sample size of women that continued smoking and alcohol consumption forms a limitation. The statistical power to demonstrate associations with continued smoking may have been limited. Further, the prevalence rates of certain events were quite low implying that our results need to be interpreted carefully, i.e. especially the positive association between crime related events and continued alcohol consumption. In addition, the results from the analyses of individual stressful events must be interpreted with some caution in view of the large number of associations tested, and thus should be interpreted in the context of the findings from the event categories. Replication by future prospective studies is essential, preferably including larger samples. Another potential limitation of our study was the use of self-report data only. Especially questions about smoking and alcohol consumption during pregnancy may be susceptible to social desirable reporting, i.e. underreporting. However, it seems unlikely that any underreporting would depend on the events experienced. Therefore, the associations were likely unaffected. Furthermore, by using a self-report questionnaire to measure stressful events, we were unable to assess the context of stressful events [57] and it may be possible that reported events overlap in occurrence (e.g. ‘having problems at work’ and ‘losing job’). Finally, we did not have data on smoking or alcohol consumption status later in pregnancy and status may have changed. Therefore, it is unclear whether our findings can be generalized to exposure to smoking and alcohol consumption beyond the period of mid-pregnancy. On the other hand, it has been shown that after the first trimester of pregnancy smoking and alcohol consumption status hardly change [58], [59]. The present study also has several strengths. First, our findings contribute to the limited literature available on psychological differences between women who continue versus quit smoking and alcohol consumption during pregnancy [60]. Second, we included stressful events that had occurred during pregnancy only. Other studies assessed stressful events ‘in the past 12 months’ thus including events before pregnancy as well [27], [61]. This may cloud associations as events in early pregnancy are rated as more stressful [62]. Moreover, including the time period before pregnancy might increase recall bias as events may be susceptible to forgetfulness with an increased time interval between occurrence of event and measurement. Our study aimed to reduce recall bias by assessing stressful events close to the period of interest.

In conclusion, this study showed that the total perceived severity of stressful events during early and mid-pregnancy increases the odds of continued alcohol consumption during mid-pregnancy. This increase concerned especially events in the categories ‘conflict with loved ones’, ‘crime related’, and ‘pregnancy-specific’. No statistically significant associations emerged between event categories and continued smoking. The impact of stressful events during early and mid-pregnancy, including pregnancy-specific events, should be identified and targeted at in healthy lifestyle strategies conducted by health care providers. Support by especially the partner and the family may act as a protective factor for maternal distress. Pregnant women and their partners and family should be assisted in acknowledging the importance of this source of support.

## Supporting Information

Table S1 **Overview of all stressful events showing the prevalence per smoking and alcohol consumption status.** The unadjusted odds ratio (OR) is given for continued versus quit use. Group severity weights per event item are based on the total study sample (n = 2287).
(DOC)
