# Supplementary material for: Stressful Events and Continued Smoking and Continued Alcohol Consumption during Mid-Pregnancy
Source: PLoS One. 2014 Jan 20;9(1):e86359. doi: 10.1371/journal.pone.0086359 (PMC3896477; doi:10.1371/journal.pone.0086359)
Supplement: Table S1 — Overview of all stressful events showing the prevalence per smoking and alcohol consumption status. The unadjusted odds ratio (OR) is given for continued versus quit use. Group severity weights per event item are based on the total study sample (n = 2287). (DOC) [file pone.0086359.s001.doc]

Table S1: Overview of all stressful events showing the prevalence per smoking and alcohol consumption status. The unadjusted odds ratio (OR) is given for continued versus quit use. Group severity weights per event item are based on the total study sample (n=2287).

|  | **Prevalence: n (%)** | | | | **Prevalence: n (%)** | | | | |
| --- | --- | --- | --- | --- | --- | --- | --- | --- | --- |
| **Stressful event item in questionnaire** | **Continued smoking (n=113)** | **Quit smoking (n=290)** | **Not smoking (n=1883)** | **OR (95% CIs) (continued vs quit)** | **Continued alcohol cons. (n=124)** | **Quit alcohol cons. (n=1403)** | **Not drinking (n=760)** | **OR (95% CIs) (continued vs quit)** | **Group severity weight (1-4)** |
| *Work or study related of self or partner* |  |  |  |  |  |  |  |  |  |
| - You had problems at work | 26 (23.0) | 55 (19.0) | 296 (15.7) | 1.3 (0.7;2.3) | 27 (21.8) | 236 (16.8) | 114 (15.0) | 1.3 (0.8;2.3) | 2.7 |
| - You lost your job | 16 (14.2) | 32 (11.0) | 107 (5.7) | 1.4 (0.6;2.9) | 12 (9.7) | 99 (7.1) | 45 (5.9) | 1.4 (0.6;3.2) | 2.7 |
| - You started a new job | 9 (8.0) | 28 (9.7) | 170 (9.0) | 0.8 (0.3;2.3) | 18 (14.5) | 136 (9.7) | 54 (7.1) | 1.5 (0.9;2.8) | 1.9 |
| - You took an important examination | 6 (5.3) | 21 (7.2) | 142 (7.5) | 0.7 (0.2;2.0) | 16 (12.9) | 107 (7.6) | 46 (6.1) | 1.7 (0.9;3.4) | 2.2 |
| - Your partner had problems at work | 20 (17.7) | 47 (16.2) | 243 (12.9) | 1.1 (0.6;2.2) | 18 (14.5) | 192 (13.7) | 100 (13.2) | 1.0 (0.6;1.9) | 2.5 |
| - Your partner lost his job | 12 (10.6) | 25 (8.6) | 87 (4.6) | 1.2 (0.5;3.0) | 6 (4.8) | 76 (5.4) | 43 (5.7) | 0.8 (0.3;2.4) | 2.8 |
| *Financial related events* |  |  |  |  |  |  |  |  |  |
| - You had a major financial problem | 16 (14.2) | 32 (11.0) | 105 (5.6) | 1.3 (0.6;2.8) | 6 (4.8) | 94 (6.7) | 52 (6.8) | 0.7 (0.2;2.1) | 3.0 |
| - Your income was reduced | 36 (31.9) | 70 (24.1) | 325 (17.3) | 1.4 (0.9;2.4) | 32 (25.8) | 263 (18.7) | 136 (17.9) | 1.5 (0.9;2.5) | 2.1 |
| *Events related to conflict with loved ones* |  |  |  |  |  |  |  |  |  |
| - You argued with your partner | 27 (23.9) | 57 (19.7) | 171 (9.1) | 1.3 (0.7;2.3) | 23 (18.5) | 151 (10.8) | 81 (10.7) | 1.8 (1.0;3.2)* | 2.7 |
| - You had arguments with your family and friends | 19 (16.8) | 51 (17.6) | 213 (11.3) | 0.9 (0.5;1.8) | 27 (21.8) | 173 (12.3) | 82 (10.8) | 2.0 (1.1;3.5)* | 2.7 |
| - Your partner went away | 4 (3.5) | 10 (3.4) | 13 (0.7) | ¥ | 3 (2.4) | 15 (1.2) | 9 (1.2) | ¥ | 3.0 |
| - You and your partner separated | 5 (4.4) | 6 (2.1) | 7 (0.4) | 2.1 (0.4;10.1) | 2 (1.6) | 8 (0.6) | 8 (1.2) | ¥ | 3.3 |
| *Events related to housing* |  |  |  |  |  |  |  |  |  |
| - You moved house | 17 (15.0) | 35 (12.1) | 179 (9.5) | 1.2 (0.6;2.7) | 17 (13.7) | 153 (10.9) | 60 (7.9) | 1.3 (0.7;2.6) | 2.0 |
| - You became homeless | 0 | 0 | 0 | na | 0 | 0 | 0 | na | na |
| *Events related to death of loved ones* |  |  |  |  |  |  |  |  |  |
| - Your partner died | 0 | 0 | 1 (0.1) | na | 0 | 1 (0.1) | 0 | na | 1.0 |
| - One of your children died | 3 (2.7) | 6 (2.1) | 16 (0.8) | ¥ | 2 (1.6) | 13 (0.9) | 10 (1.3) | ¥ | 4.0 |
| - One of your parents died | 5 (4.4) | 8 (2.8) | 26 (1.4) | 1.7 (0.5;6.2) | 3 (2.4) | 20 (1.4) | 16 (2.1) | ¥ | 3.9 |
| - One of your brothers or sisters died | 4 (3.5) | 3 (1.0) | 3 (0.2) | ¥ | 2 (1.6) | 6 (0.4) | 2 (0.3) | ¥ | 3.4 |
| - A friend of other relative died | 17 (15.0) | 35 (12.1) | 242 (12.9) | 1.3 (0.6;2.8) | 14 (11.3) | 171 (12.2) | 110 (14.5) | 0.9 (0.4;1.7) | 2.8 |
| *Events related to illness of self or loved ones* |  |  |  |  |  |  |  |  |  |
| - Your partner was ill | 5 (4.4) | 13 (4.5) | 47 (2.5) | 0.9 (0.2;3.5) | 3 (2.4) | 41 (2.9) | 21 (2.8) | ¥ | 2.9 |
| - One of your children was ill | 6 (5.3) | 9 (3.1) | 45 (2.4) | 2.0 (0.6;6.4) | 3 (2.4) | 35 (2.5) | 22 (2.9) | ¥ | 3.2 |
| - One of your parents was ill | 19 (16.8) | 27 (9.3) | 191 (10.1) | 1.9 (0.9;4.1) | 12 (9.7) | 160 (11.4) | 65 (8.6) | 0.8 (0.4;1.8) | 3.2 |
| - One of your brothers or sisters was ill | 3 (2.7) | 7 (2.4) | 25 (1.3) | ¥ | 3 (2.4) | 21 (1.5) | 11 (1.4) | ¥ | 3.0 |
| - A friend or other relative was ill | 12 (10.6) | 35 (12.1) | 222 (11.8) | 0.8 (0.4;1.9) | 11 (8.9) | 166 (11.8) | 93 (12.2) | 0.7 (0.3;1.5) | 2.9 |
| - You were very ill | 3 (2.7) | 11 (3.8) | 42 (2.2) | ¥ | 4 (3.2) | 31 (2.2) | 20 (2.6) | ¥ | 3.2 |
| - You were admitted to hospital | 4 (3.5) | 14 (4.8) | 77 (4.1) | ¥ | 5 (4.0) | 51 (3.6) | 40 (5.3) | 1.1 (0.4;3.3) | 2.9 |
| - You attempted suicide | 0 | 1 (0.3) | 0 | na | 0 | 1 (0.1) | 0 | na | 2.0 |
| *Events related to domestic violence or abuse* |  |  |  |  |  |  |  |  |  |
| - Your partner hurt you physically | 0 | 3 (1.0) | 3 (0.2) | na | 0 | 4 (0.3) | 2 (0.3) | na | 2.5 |
| - Your partner hurt your children physically | 0 | 0 | 4 (0.2) | na | 0 | 2 (0.1) | 2 (0.3) | na | 1.3 |
| - Your partner was emotionally cruel to you | 6 (5.3) | 13 (4.5) | 31 (1.6) | 1.1 (0.4;3.6) | 4 (3.2) | 27 (1.9) | 19 (2.5) | ¥ | 3.0 |
| - Your partner was emotionally cruel to your children | 0 | 0 | 3 (0.2) | na | 0 | 2 (0.1) | 2 (0.3) | na | 3.0 |
| - There was alcohol or drug abuse within your family or relationship | 1 (0.9) | 3 (1.0) | 7 (0.4) | ¥ | 0 | 7 (0.5) | 3 (0.4) | na | 2.9 |
| *Crime related events* |  |  |  |  |  |  |  |  |  |
| - Your house or car was burgled | 4 (3.5) | 10 (3.4) | 31 (1.6) | ¥ | 3 (2.4) | 35 (2.5) | 8 (1.2) | ¥ | 2.1 |
| - You were involved in an accident | 1 (0.9) | 11 (3.8) | 42 (2.2) | ¥ | 6 (4.8) | 31 (2.2) | 17 (2.2) | 2.2 (0.8;6.3) | 2.3 |
| - You were in trouble with the law | 1 (0.9) | 0 | 3 (0.2) | na | 0 | 3 (0.2) | 2 (0.3) | na | 2.1 |
| - Your partner was in trouble with the law | 2 (1.8) | 5 (1.7) | 10 (0.5) | ¥ | 3 (2.4) | 8 (0.6) | 5 (0.6) | ¥ | 2.6 |
| - You were convicted for an offence | 0 | 0 | 0 | na | 0 | 0 | 0 | na | Na |
| - You were a victim of an offence | 2 (1.8) | 3 (1.0) | 9 (0.5) | ¥ | 4 (3.2) | 7 (0.5) | 3 (0.4) | ¥ | 3.1 |
| - You were a victim of sexual abuse | 0 | 7 (2.4) | 8 (0.4) | na | 0 | 9 (0.6) | 6 (0.8) | na | 3.4 |
| *Pregnancy-specific events* |  |  |  |  |  |  |  |  |  |
| - Your pregnancy was unwanted | 12 (10.6) | 22 (7.6) | 33 (1.2) | 1.4 (0.6;3.3) | 9 (7.3) | 37 (2.6) | 20 (2.6) | 2.7 (1.1;6.7)* | 2.6 |
| - You tried to have an abortion | 3 (2.7) | 8 (2.8) | 11 (0.6) | ¥ | 3 (2.4) | 10 (0.7) | 8 (1.2) | ¥ | 3.5 |
| - You found that your partner did not want your child | 4 (3.5) | 9 (3.1) | 2 (0.1) | ¥ | 1 (0.8) | 9 (0.6) | 5 (0.6) | ¥ | 3.0 |
| - You were bleeding and thought you might miscarry | 20 (17.7) | 52 (17.9) | 301 (16.0) | 1.0 (0.5;1.9) | 26 (21.0) | 226 (16.1) | 121 (15.9) | 1.4 (0.8;2.3) | 3.3 |
| - You had a test to see if your baby might not be normal | 37 (32.7) | 107 (36.9) | 742 (39.4) | 0.8 (0.5;1.5) | 65 (52.4) | 568 (40.5) | 253 (33.3) | 1.6 (1.0;2.5)* | 2.2 |
| - You had a result on a test that suggested your baby might not be normal | 4 (3.5) | 10 (3.4) | 59 (3.1) | ¥ | 5 (4.0) | 38 (2.7) | 30 (3.9) | 1.4 (0.5;4.5) | 3.3 |
| - You were told that you were going to have twins | 3 (2.7) | 5 (1.7) | 59 (3.1) | ¥ | 4 (3.2) | 35 (2.5) | 27 (3.6) | ¥ | 2.9 |
| - You heard something that had happened might be harmful to the baby | 6 (5.3) | 9 (3.1) | 83 (4.4) | 1.8 (0.5;6.2) | 8 (6.5) | 57 (4.1) | 33 (4.3) | 1.7 (0.7;4.3) | 3.0 |

Note: for some variables numbers do not add to the total due to rounding of imputed values

¥: Due to low numbers in cells no value is presented

* p<0.05
